# Image Quality and Radiation Dose of Lower Extremity CT Angiography Using 70 kVp, High Pitch Acquisition and Sinogram-Affirmed Iterative Reconstruction

**DOI:** 10.1371/journal.pone.0099112

**Published:** 2014-06-10

**Authors:** Li Qi, Felix G. Meinel, Chang Sheng Zhou, Yan E. Zhao, U. Joseph Schoepf, Long Jiang Zhang, Guang Ming Lu

**Affiliations:** 1 Department of Medical Imaging, Jinling Hospital, Clinical School of South Medical University, Nanjing, Jiangsu, China; 2 Department of Radiology and Radiological Science, Medical University of South Carolina, Charleston, South Carolina, United States of America; Northwestern University Feinberg School of Medicine, United States of America

## Abstract

**Objectives:**

The purpose of this study was to assess image quality and radiation dose of lower extremity CT angiography (CTA) with 70 kVp, high pitch acquisition and sinogram-affirmed iterative reconstruction (SAFIRE).

**Methods:**

Lower extremity CTAs were performed on 44 patients: 22 patients were examined using protocol A (120 kVp, pitch of 0.85 and 120 ml of contrast agent on a first-generation dual-source CT) (120 kVp group) and 22 patients were evaluated with protocol B (70 kVp, pitch of 2.2 and 80 ml of contrast agent on a second-generation dual-source CT) (70 kVp group). Images from the 120 kVp group were reconstructed with filtered back projection (FBP) and images from the 70 kVp group with SAFIRE. The attenuation, image noise, signal-to-noise ratio (SNR) and contrast-to-noise ratio (CNR) were calculated. Two radiologists subjectively assessed image quality of lower extremity arteries, plantar arterial enhancement and venous contamination of all patients. Radiation dose was compared between the two groups.

**Results:**

Higher mean intravascular attenuation was obtained in the 70 kVp group (70 vs. 120 kVp group, 555.4±83.4 HU vs. 300.9±81.4 HU, P<0.001), as well as image noise (20.0±2.8 HU vs. 17.5±3.2 HU, P = 0.010), SNR (32.0±7.0 vs. 19.1±6.9, P<0.001) and CNR (28.1±6.6 vs 15.9±6.3, P<0.001). No difference in subjective image quality and plantar arterial enhancement was found between 120 kVp group and 70 kVp group (all P>0.05). The venous contamination score was 1.5±0.8 for 120 kVp group while no venous contamination was found in 70 kVp group. The inter-observer agreement was moderate to good for both groups (0.515∼1, P<0.001). The effective dose was lower in 70 kVp group (0.3±0.1 mSv) than in 120 kVp group (1.6±0.7 mSv)(*P*<0.001).

**Conclusions:**

Lower extremity CTA using 70 kVp, high pitch acquisition and SAFIRE, except increasing imaging noise, allows for lower radiation dose and contrast material volume without compromising image quality.

## Introduction

Peripheral arterial disease (PAD) is a common, chronic and progressive health problem. It is reported that the overall prevalence of PAD was 16.7% in Chinese patients with type 2 diabetes and 27.5% in Chinese patients with hypertension [1], [2]. The mortality rates in patients with PAD average 2% per year and the rates of nonfatal myocardial infarction, stroke, and vascular death are 5% to 7% per year [3]. Early diagnosis and appropriate medical interventions can mitigate limb-specific symptoms, improve quality of life, and decrease systemic cardiovascular risk [4].

CT angiography (CTA), as a non-invasive and safe examination, has been widely used in clinical practice to diagnose PAD and acute lower-extremity vascular injury with high accuracy [5]–[8]. However, the radiation exposure to the patients and the relatively high dose of contrast medium necessary to perform lower extremity CTA due to the wide scan range from the abdomen to the feet have raised concerns. A previous study has shown that the weighted CT dose index (CTDIw) of lower extremity CTA could be as high as 12.2 mGy and the volume of contrast material could be as much as 140 ml [5]. With the rapid refinement of CT technology, various strategies of reducing radiation dose and dosage of contrast medium have been developed. The technique of lowering tube voltage is most often used among various dose-reducing strategies. The tube voltage of 80 kVp has been verified to be feasible for lower extremity CTA with substantial reduction of radiation dose [9], [10]. Most recently, Duan et al have reduced radiation dose to 1.94 mSv and contrast medium volume to 1.2 mL/kg when a tube voltage of 70 kVp was used in lower extremity CTA [11]. However, further reducing radiation dose of low extremity CTA appears to be feasible. For example, high pitch CTA acquisition has been used to evaluate deep vein thrombosis [12]. The combination of 70 kVp and high-pitch acquisition technique holds potential to further reduce radiation dose. In addition, sinogram-affirmed iterative reconstruction (SAFIRE), a raw-data-based IR algorithm, has the potential to improve image quality and reduce image noise which benefits for the radiation dose saving [13]. However, to the best of our knowledge, no study has been performed on lower extremity CTA using 70 kVp combined with high pitch acquisition and iterative reconstruction. The aim of our study was therefore to investigate the feasibility, image quality and radiation dose of lower extremity CTA using 70 kVp combined with dual-source high pitch acquisition and iterative reconstruction.

## Materials and Methods

### Study subjects

This retrospective study was approved by Jinling Hospital, Clinical School of South Medical University, Nanjing, China and informed consent was waived. The inclusion criteria for the enrollment was the utilization of the two special scan protocols (120 kVp scan protocol and 70 kVp, high-pitch scan protocol) for lower extremity CTA. Subjects were excluded when severe beam hardening artifacts were present because of metal stents. Finally, a total of 44 patients (32 male and 12 female; mean age 60±20 years; age range 19–90 years) undergoing lower extremity CTA because of known tumors (*n* = 5), injury (*n* = 12), or suspected arterial occlusive disease (*n* = 27) were included in the study.

Among these patients, 22 patients (16 male and 6 female; mean age 56±22 years; age range 19–90 years) who had undergone CTA of the lower extremities at 120 kVp on a first-generation dual-source CT system between December 2012 and November 2013 were selected as the control (120 kVp group). Of these 22 patients, one patient had soft tissue tumors, 8 patients had traumatic lower extremity injury and 13 patients had peripheral artery disease.

Another 22 patients (16 male and 6 female; mean age 63±18 years; age range 20–86 years) underwent lower extremity CTA using 70 kVp combined with high pitch (2.2) acquisition and iterative reconstruction on a second-generation dual-source CT system (70 kVp group). These studies were performed between July 2013 and October 2013 because of known tumors (*n* = 4), traumatic lower extremity injury (*n* = 4) and suspected peripheral artery disease (*n* = 14).

### CT protocol and contrast medium infusion protocols

All patients in the 120 kVp group were examined on a first-generation dual-source CT (Somatom Definition; Siemens Medical Solutions, Germany). For the patients in the 70 kVp group, a second-generation dual-source CT system (Definition Flash, Siemens Medical Solutions, Forchheim, Germany) with an integrated circuit detector was used. The acquisition parameters in the 120 kVp group were tube voltage, 120 kVp; pitch, 0.85; collimation, 64×0.6 mm; slice thickness, 1.0 mm; reconstruction increment, 0.7 mm. The acquisition parameters in the 70 kVp group were tube voltage, 70 kVp; pitch, 2.2; collimation, 128×0.6 mm; slice thickness, 1.0 mm; reconstruction increment, 1 mm. Automatic tube current modulation (ATCM, CARE Dose 4D; Siemens Medical Solutions, Germany) was used in both acquisitions with a quality reference tube current-time product of 150 mAs for the 120 kVp group and a reference tube voltage of 100 kVp and a reference tube current-time product of 370 mAs for the 70 kVp group. The mean acquisition time was recorded for each patient. Each acquisition was performed in the craniocaudal direction from the abdominal aortic bifurcation to the feet.

In all patients, iopromide (300 mg I/mL, Bayer Schering, Berlin, Germany) was delivered via an 18-gauge intravenous cannula placed into a superficial vein positioned in the antecubital fossa. Contrast enhancement was achieved by injecting 120 mL of contrast material (first 80 mL at 4 mL/s and then 40 mL at 2 mL/s) for the 120 kVp group and 80 mL (first 60 mL at 4 mL/s and then 20 mL at 2 mL/s) for the 70 kVp group, followed by a 30 mL saline flush at 2 mL/s, which were designed according to our clinical experience. The bolus-tracking technique was used in both groups. In the 120 kVp group, a region of interest (ROI) was positioned in the aortic bifurcation and image acquisition automatically started 14 seconds after the signal attenuation of ROI reached the predefined threshold of 100 Hounsfield Units (HU). In order to adapt timing to the high-pitch scanning, image acquisition in 70 kVp group started with a 6-second delay after the signal attenuation of the proximal popliteal artery rather than the abdominal aortic bifurcation had reached the predefined threshold of 100 HU.

### Image reconstruction

CT data from the 120 kVp group was constructed with filtered back projection (FBP, Siemens Healthcare, Forchheim, Germany), and data from the 70 kVp group was constructed with sinogram-affirmed iterative reconstruction (SAFIRE, Siemens Healthcare, Forchheim, Germany). For the SAFIRE algorithm, five presets (strength 1–5) are available for adapting the noise model and controlling image impression and noise reduction [14]. As recommended by the manufacturer, a medium strength level of 3 was used.

### Image analysis

All data sets were transferred to a dedicated workstation (Syngo.via; Siemens Medical Solutions, Forchheim, Germany). The anteroposterior and transverse diameters of each patient were measured at the level of the aortic bifurcation on axial CT images as a metric of body habitus. Effective diameter, defined as the square root of the anteroposterior diameter times the transverse diameter, was calculated for each patient. Maximum intensity projections (MIP) and curved planar reformations (CPR) were generated for each patient. To evaluate objective image quality, the intravascular attenuation was measured in five locations (aortic bifurcation, iliac bifurcation, proximal and middle femoral artery and proximal popliteal artery) on axial CT images. The measurements were averaged to derive a mean intravascular attenuation. Moreover, the attenuation of the muscle and the noise within subcutaneous fat (standard deviation of the CT attenuation) at the same slice of the measurement of intravascular attenuation of the five locations were also measured to calculate signal-to-noise ratio (SNR) and contrast-to-noise ratio (CNR) of each arterial segment. The image noise was defined as the mean value of the five SDs in subcutaneous fat. The attenuations were measured in a region of interest (ROI) within the vessels which was defined to be as large as possible while avoiding calcifications, plaques and stenoses. The SNR and CNR were calculated as follows:

SNR  =  attenuation of the vessel lumen/SD of subcutaneous fat

CNR  =  (attenuation of the vessel lumen - attenuation of muscle)/SD of subcutaneous fat

In order to evaluate subjective image quality, all CT images (axial images, MIP and CPR images) were independently reviewed by two radiologists (with 5 and 2 years of reading experience, respectively) blinded to patients' information and CTA protocols. Before image evaluation, the observers were instructed to divide vascular structures into three segments (the aorto-iliac area, the femoro-popliteal region, and the lower leg segment) and to then score the image quality in these segments using a 4-point scale according to the degree of noise artifacts, the presence of streak artifacts and graininess of the images. The scoring criteria were as follows [15]: Poor (grade 1): graininess or streak artifacts were significant and did not provide sufficient information for the diagnosis; Adequate (grade 2): the examination provided acceptable information but unsatisfactory image quality; Good (grade 3): image quality was satisfactory enough to provide the information necessary to make an adequate radiological diagnosis; Excellent (grade 4): image quality provided optimal information for a radiological diagnosis. In addition, because the plantar vessels were too small to measure their attenuations, the enhancement of plantar arteries was subjectively scored using a 4-point scale: 1, not visible; 2, poor, visible but insufficient arterial enhancement for diagnosis; 3, moderate, enough arterial enhancement for diagnosis; 4, optimal arterial enhancement for diagnosis. Venous contamination was also graded on a three-point scale: 1, not visible; 2, visible but does not affect diagnostic interpretation; 3, present and definitely compromises diagnostic interpretation. In the event of observer disagreement, another reading session was convoked to reach an agreement.

### Radiation dose estimation

For an estimation of the radiation dose, the volume CT dose index (CTDIvol) and the dose length product (DLP) of each patient were recorded. We also calculated the effective radiation dose delivered for the pelvic and hip regions, since the exposure of the extremities contributes only minimally to the overall effective dose and the conversion factors for legs are not available [11], [16]. The distance from the pelvic crest to the proximal third of the thighs, including the testicles in men and vulva in women, was measured in each patient. The effective dose (ED) was calculated by multiplying the product of CTDIvol and the distance of the pelvic crest to the proximal third of the thighs by a conversion factors of 0.015 [17]. In addition, the acquisition length of the abdominal aorta and runoff arteries was calculated by dividing the DLP by the CTDIvol.

### Statistical analysis

Statistical analyses were performed using the SPSS software version 16.0 (SPSS Inc. Chicago, IL, USA). Quantitative variables were expressed as mean ± SD, while categorical variables were expressed as frequencies or percentages. The Mann-Whitney test was used to compare categorical characteristics and the independent-sample t test was used to compare continuous variables. Kappa analysis was used to assess the inter-observer agreement of the subjective image quality rating. A k value of less than 0.20 indicates poor agreement; a k value of 0.21–0.40, fair agreement; a k value of 0.41–0.60, moderate agreement; a k value of 0.61–0.80, good agreement; and a k value of 0.81–1.00, very good agreement. P<0.05 was considered to indicate a statistically significant difference.

## Result

There were no statistically significant differences in age (56±22 years vs. 63±18 years, *P* = 0.216), sex (male, 72.7% vs. 71.4%, *P* = 0.925), anteroposterior body diameter (18.8±2.5 cm vs. 19.8±2.7 cm, P = 0.297), transverse diameter (31.2±2.8 cm vs. 30.3±2.8 cm, P = 0.218), effective diameter (24.1±2.4 cm vs. 24.4±2.6 cm, P = 0.708) and acquisition range (108.6±10.9 cm vs. 113.1±5.8 cm, *P* = 0.010) between the 120 kVp group and the 70 kVp group. The mean acquisition time was 3.8 seconds in the 70 kVp group and 32.9 seconds in 120 kVp group (P<0.001).

The attenuations measured in all above-mentioned locations were shown in Table 1. The mean intravascular attenuation was 300.9±81.4 HU for the 120 kVp group and 555.4±83.4 HU for the 70 kVp group (P<0.001), indicating an 84.6% increase in the attenuation. Furthermore, the image noise in the 120 kVp group was lower than that in the 70 kVp group (17.5±3.2 HU vs. 20.0±2.8 HU, respectively, P = 0.010), as were the SNR (19.1±6.9 vs. 32.0±7.0, respectively, P<0.001) and CNR (15.9±6.3 vs. 28.1±6.6, respectively, P<0.001).

**Table 1:** Objective image quality evaluation of lower extremity CTA.

Parameter	120 kVp group	70 kVp group	*P* value
Aortic bifurcation			
Mean CT value (HU)	312.9±84.9	504.7±102.7	<0.001
SNR	17.1±8.1	22.5±8.6	0.040
CNR	14.5±7.6	19.6±8.0	0.038
Iliac bifurcation			
Mean CT value (HU)	298.8±75.0	504.61±100.0	<0.001
SNR	17.7±6.6	24.4±8.3	0.005
CNR	14.8±6.1	21.3±7.8	0.004
Proximal femoral artery			
Mean CT value (HU)	295.2±82.8	544.0±87.9	<0.001
SNR	16.1±8.1	28.5±9.3	<0.001
CNR	13.7±7.5	25.1±8.8	<0.001
Middle femoral artery			
Mean CT value (HU)	299.3±88.9	578.7±90.4	<0.001
SNR	23.6±13.3	35.3±11.8	0.004
CNR	19.7±11.8	31.2±11.0	0.002
Proximal popliteal artery			
Mean CT value (HU)	292.9±70.3	620.3±100.3	<0.001
SNR	23.8±10.9	47.0±16.0	<0.001
CNR	19.6±9.6	41.5±14.6	<0.001
Mean			
Mean CT value (HU)	300.9±81.4	555.4±83.4	<0.001
SNR	19.1±6.9	32.0±7.0	<0.001
CNR	15.9±6.3	28.1±6.6	<0.001
Noise (HU)	17.5±3.2	20.0±2.8	0.010

SNR = signal-to-noise ratio; CNR = contrast-to-noise ratio.

**Figure 1:**
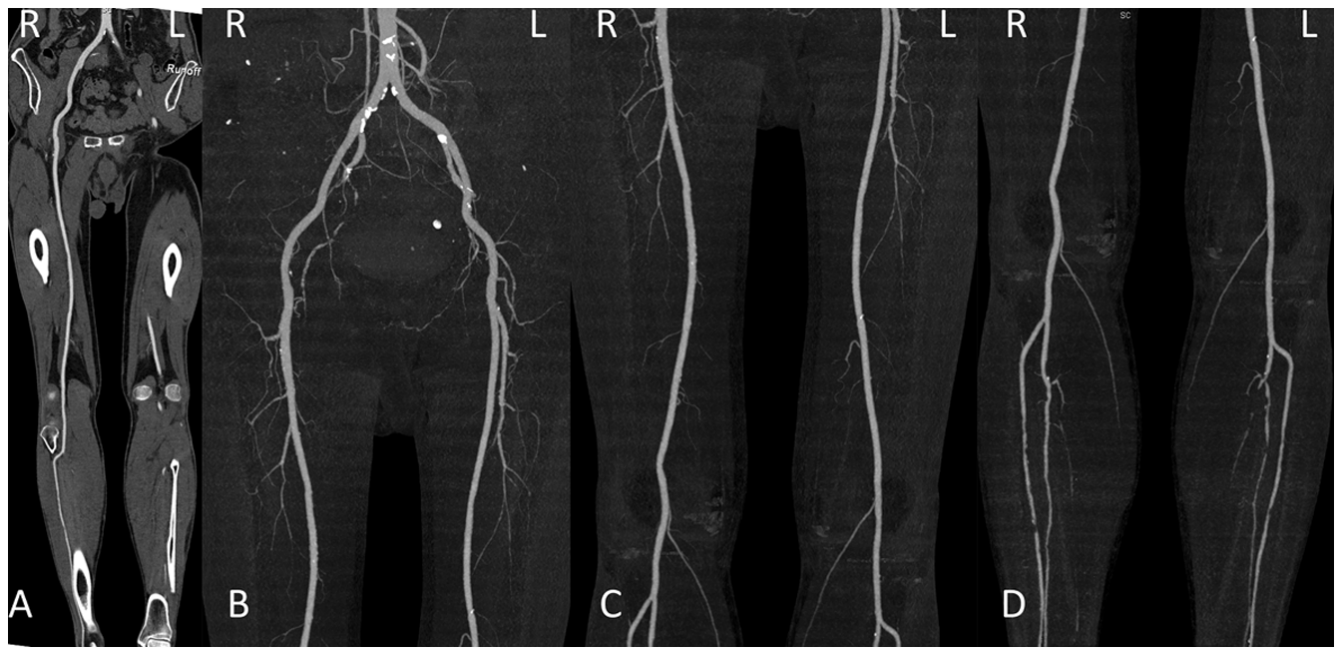
Lower extremity CTA using 120-year-old man with PAD. A, curved planar reformatted image and B–D, maximum intensity projection images. The image quality was visually classified as score 4 (excellent) in the aortoiliac (B), femoropopliteal (C) and lower leg segments (D).

**Figure 2:**
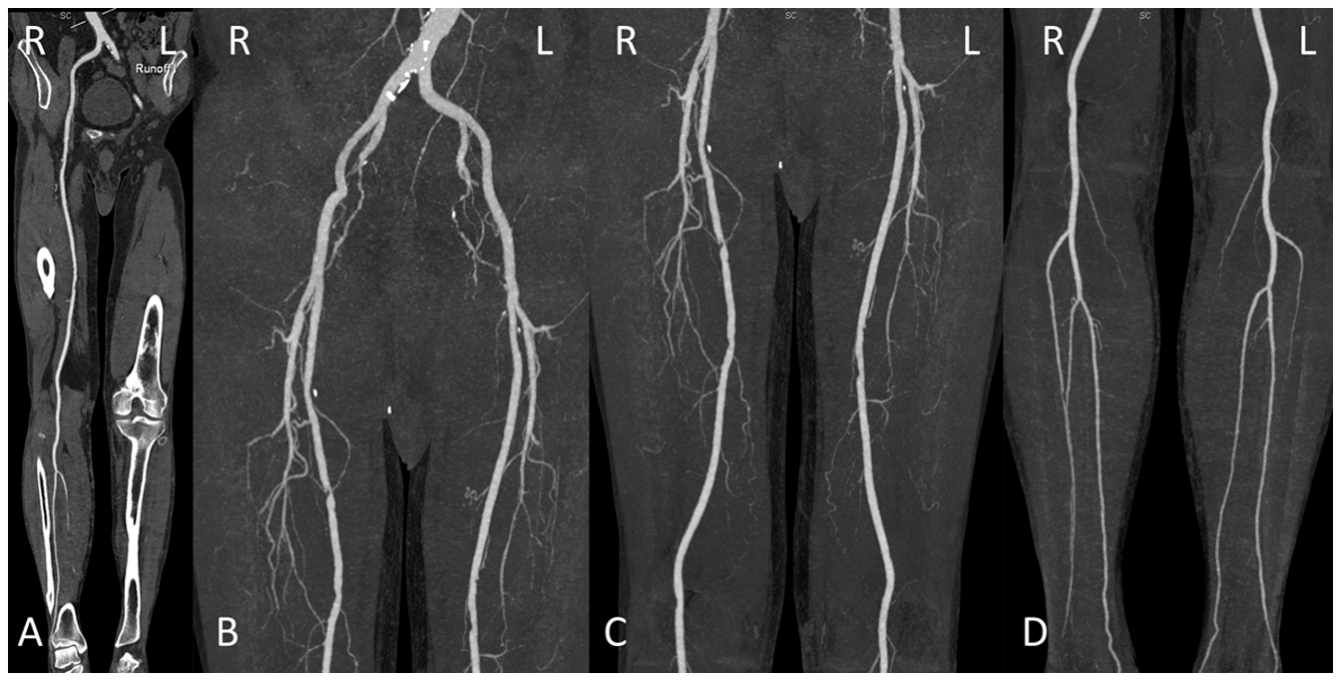
Lower extremity CTA using 70-year-old man with PAD. A, curved planar reformatted image and B–D, maximum intensity projection images. The image quality was visually classified as score 4 (excellent) in the aortoiliac (B), femoropopliteal (C) and lower leg segments (D).

**Figure 3:**
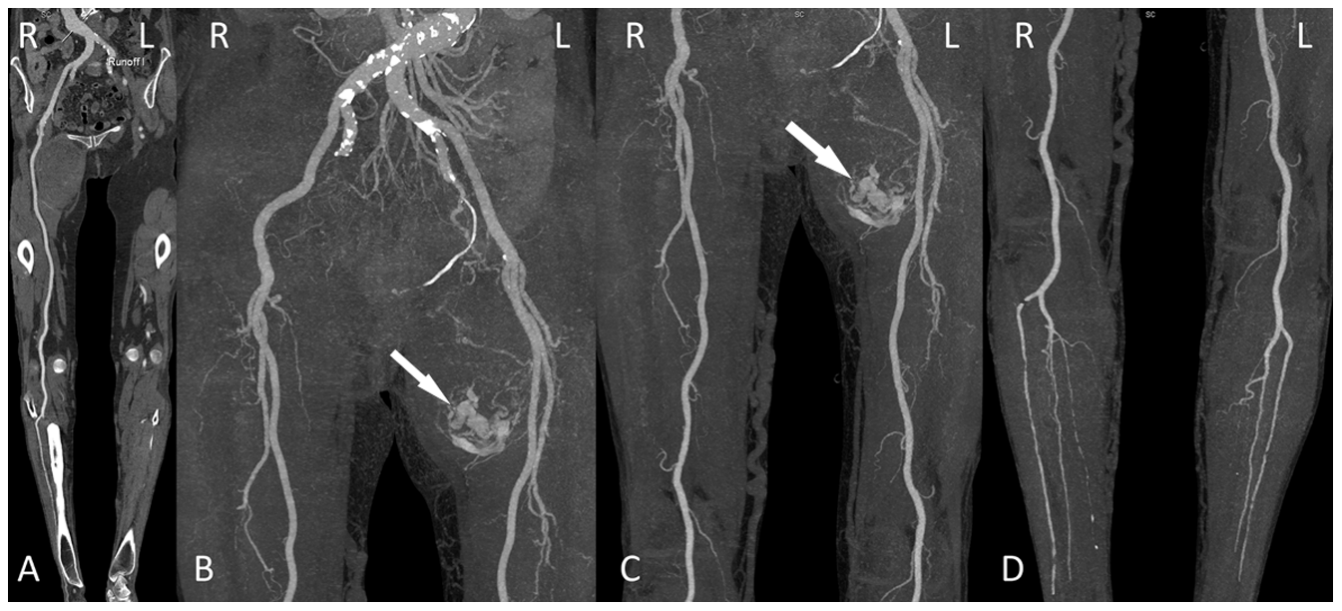
Lower extremity CTA using 70-year-old woman with tumor. A, curved planar reformatted image and B–D, maximum intensity projection images. The image quality was visually classified as score 3 (good) in the aortoiliac (B), femoropopliteal (C) and lower leg segments (D). Note abnormal hypervascular mass (myxofibrosarcoma) in the left thigh (arrow in panels B and C).

**Figure 4:**
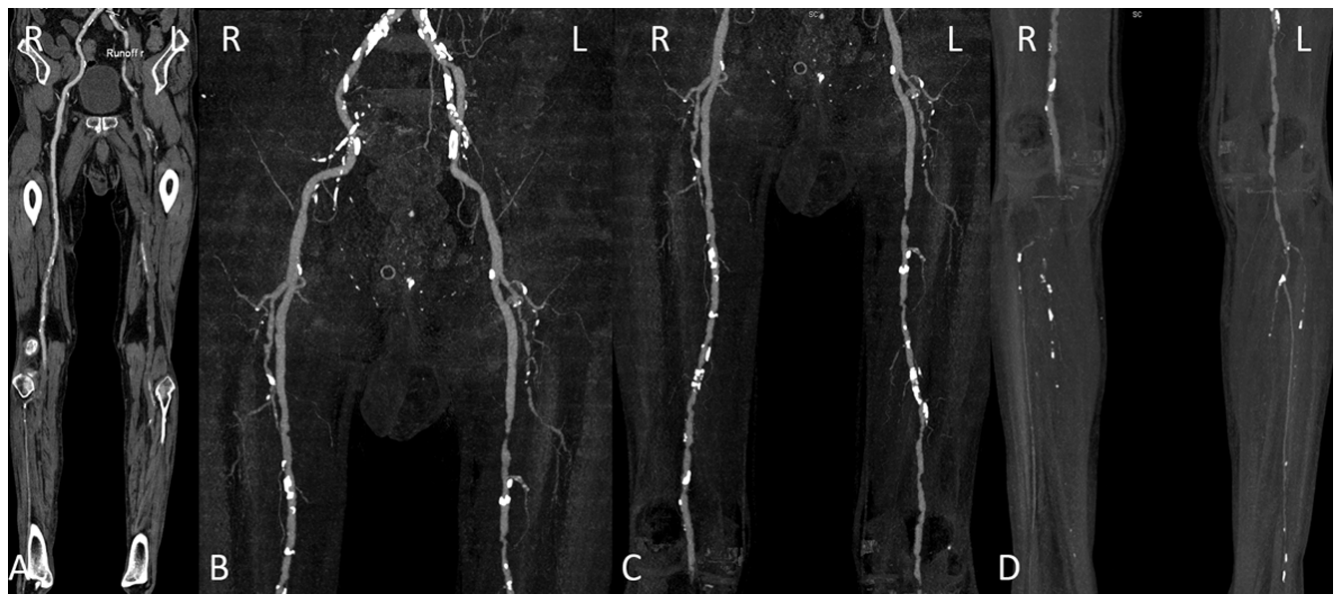
Lower extremity CTA using 120-year-old man with PAD. A, curved planar reformatted image and B–D, maximum intensity projection images. The image quality was visually classified as score 3 (good) in the aortoiliac (a) and femoropopliteal (b) and score 2 (adequate) in the lower leg segments (c) because of poor visualization. Note extensive calcification of bilateral arteries of low extremity.

A total of 254 segments in 44 patients were assessed for subjective image quality, while 10 segments were excluded because of vascular occlusion. The kappa values for image quality, enhancement of plantar arteries and venous contamination were 0.605, 0.672, 0.707 for 120 kVp group and 0.690, 0.515, 1 for 70 kVp group (all P<0.001). There was found no significant difference in the image quality score (120 vs. 70 kVp group, 3.5±0.5 vs. 3.5±0.4, P = 0.981) and plantar arterial enhancement score (120 vs. 70 kVp group, 2.9±0.6, 2.7±1.1, P = 0.576) between the two groups. The image quality scores of the three segments were displayed in Table 2. Figures 1–4 show some representative cases with different scores using 120 kVp and 70 kVp protocols. Venous contamination was not found in any patient of 70 kVp group. The venous contamination score was 1.5±0.8 for 120 kVp group.

**Table 2:** Subjective image quality scores of three arterial segments of the lower extremities.

Score	120 kVp group	70 kVp group	*P* value
Aorto-iliac area			
Reader 1	3.6±0.5	3.7±0.5	0.366
Reader 2	3.6±0.5	3.5±0.6	0.766
Both	3.6±0.5	3.7±0.5	0.598
Femoro-popliteal region			
Reader 1	3.7±0.5	3.6±0.5	0.511
Reader 2	3.7±0.5	3.6±0.6	0.629
Both	3.6±0.6	3.7±0.5	0.762
lower leg segment			
Reader 1	3.1±0.7	3.4±0.6	0.156
Reader 2	3.1±0.5	3.3±0.8	0.140
Both	3.3±0.7	3.1±0.6	0.316
Mean			
Reader 1	3.5±0.4	3.6±0.4	0.313
Reader 2	3.5±0.4	3.5±0.5	0.391
Both	3.5±0.5	3.5±0.4	0.981

The radiation dose estimation for both groups was summarized in Table 3. The DLP and CTDIvol of the abdominal aorta and runoff arteries were significantly lower in 70 kVp group when compared with the 120 kVp group (P<0.001). The mean distance from pelvic crest to the proximal third of the thighs was 26.9±2.9 cm for 120 kVp group and 26.4±2.0 cm for 70 kVp group (P = 0.509). The mean DLP of the distance from pelvic crest to the proximal third of the thighs was 109.4±47.7 mGy×cm for 120 kVp group and 19.5±2.1 mGy×cm for 70 kVp group, resulting in a mean ED of 1.6±0.7 in 120 kVp group and 0.3±0.1 mSv in 70 kVp group.

**Table 3:** Radiation dose comparison of two lower extremity CTA protocols.

Parameters	120 kVp group	70 kVp group	*P* value
DLP (mGy×cm)	434.5±164.1	83.7±7.4	<0.001
CTDIvol (mGy)	4.0±1.4	0.7±0.1	<0.001
ED (mGy)	1.6±0.7	0.3±0.1	<0.001

DLP = dose-length product; CTDIvol = volume CT dose index; ED = effective dose.

## Discussion

The present study demonstrates the feasibility of lower extremity CTA using 70 kVp combined with high pitch acquisition and iterative reconstruction on a second-generation dual-source CT system with an integrated circuit detector. Moreover, this protocol resulted in an 81.3% radiation dose reduction and a 33.3% decrease in the volume of contrast material compared to our standard protocol with 120 kVp on a first-generation dual-source CT without compromising image quality.

Lowering tube voltage is an effective method to reduce radiation dose because of the exponential relationship between kilovoltage and radiation dose [18]. Lowering tube voltage from 120 kVp to 80 kVp has been reported to result in a 30% reduction in radiation dose for lower extremity CTA [9]. In our study, a tube voltage of 70 kVp was used, which has been verified to be feasible and accurate in Duan's study [11]. In addition, increasing pitch is another method to reduce radiation dose by shortening image acquisition time [19]. Amacker et al reported that the mean radiation dose decreased by 20% and 42% when the pitch was increased from 1 to 3.2 for routine chest and abdominal CT scans [20]. The two strategies have been widely used in combination for coronary CTA [21], [22] but rarely in lower extremity CTA. Our study applied the two radiation dose reducing strategies to lower extremity CTA and revealed that the radiation dose of lower extremity CTA could be reduced by 81.3% with the 70 kVp high-pitch CTA protocol. The radiation dose of our low extremity CTA is lower than that of Duan's study using 70 kVp for low extremity CTA (1.94 mSv) [11].

Moreover, lowering tube voltage and raising pitch are not only the strategies to reduce radiation dose but also the strategies to reduce contrast material requirements. Lowering tube voltage effectively increases attenuation of the iodinated contrast agent, which offers a potential of reducing the dose of iodine. It has been reported that a reduction of tube voltage from 120 kVp to 80 kVp allows reducing the volume of contrast medium from 1.8 mL/kg to 1.2 mL/kg without deterioration of vascular enhancement for lower extremity CTA [9]. Additional, long scan time of lower extremity CTA requires high dose of contrast medium injection to ensure the contrast enhancement peak is high and wide enough. However, the shortened acquisition time in high-pitch acquisition requires only a short temporal window of vascular enhancement and the CT acquisition can “ride the bolus”, which can reduce the volume of contrast material. Yang et al reported that dual-source CT coronary angiography using a pitch of 3.4 can be performed with 30 ml of contrast medium without affecting vessel enhancement [23]. In combination with lowering tube voltage and high pith, lower extremity CTA can be performed with 80 mL of contrast material with a mean CT value of 555.4 HU, which was statistically higher than that of standard 120 kVp group. Our finding of obtaining more than 500 HU of peripheral arteries when using 70 kVp and the high pitch technique indicates the possibility of further lowering the contrast material volume in this CTA protocol. However, for the long scan range of lower extremity CTA, pitch affect the enhancement of distal vessels. In our study, we choose the proximal popliteal artery rather than the abdominal aortic bifurcation as the monitoring position to adapt the high-pitch scan of 70 kVp group, which resulted in no difference in the plantar arterial enhancement when compared with 120 kVp group. Additional, no venous contamination was found in patient of 70 kVp group.

However, the major disadvantage of both strategies for reducing radiation dose and contrast material is the increase in image noise, which may impair diagnostic interpretability. To counteract this problem, sinogram-affirmed iterative reconstruction (SAFIRE) was used in the 70 kVp group to reduce image noise. SAFIRE is a noise-modeling technique which compares reconstructed and measured data in the raw data domain and iteratively corrects the images to reduce noise and improve image quality [24], [25]. Several studies have shown the benefit of SAFIRE algorithm for various clinical applications [26]–[28]. Han has reported that low-dose pediatric cardiac CT images constructed with SAFIRE had a 34% decrease in noise when compared with images constructed with the standard weighted filtered back projection [26]. Besides, the new integrated circuit detector used in the second-generation 128-section dual-source CT system (SOMATOM Definition Flash with an integrated circuit detector; Siemens Healthcare, Forchheim, Germany) has the capability to reduce electronic noise and improve spatial resolution compared with previous detector technology. Spiczak et al have demonstrated that a significantly lower image noise was observed in data sets scanned with integrated detector compared to data sets scanned with conventional detector [29]. Although these two strategies were used to decrease noise in the 70 kVp group, the image noise in the 70 kVp group was still higher than in the 120 kVp group. Baumueller's study [30] showed that image noise at 80 kVp reconstructed with SAFIRE was significantly lower than that in datasets acquired at 100 kVp and reconstructed with FBP. The difference probably resulted from the more pronounced reduction in tube potential and the simultaneous increase in pitch in our study. Another possible interpretation is that the strength level of 3 for SAFIRE may not be sufficient for 70 kVp data compared with 80 kVp data, this hypothesis merits further studies. Although the image noise in the 70 kVp group was higher than that in the 120 kVp group, the image quality was still diagnostic with a mean subjective image quality score of 3.5. No statistically significant difference was found for subjective image quality evaluation between the two groups.

There are certain limitations to our study that must be acknowledged. First, patients' body mass index was not compared between the two groups because the height and weight were not recorded when patients underwent CTA examinations and were thus not available in this retrospective study. However, as an alternative anthropometric parameter to gauge body habitus, we measured the anteroposterior and transverse diameter in the CT images and calculated effective diameter of each study subject, which showed no significant difference between the two groups. Second, because of the retrospective design of this study, it was impossible to exactly control several parameters influencing radiation dose and image quality, such as the collimation. Additionally, selection bias because of the nature of retrospective study may occur in our study. Therefore, a prospective study is required. Third, a relatively small sample size of 44 patients was investigated in this feasibility study. A further study with a larger sample should be performed to confirm the results of our single-center study. Fourth, the diagnostic accuracy of lower extremity CTA using 70 kVp and high pitch acquisition was not assessed, since in most patients invasive angiography as the reference standard was not available. A further study assessing the diagnostic accuracy of lower extremity CTA using this protocol is required.

In conclusion, lower extremity CTA using 70 kVp, high pith acquisition and iterative reconstruction is feasible and allows for lowering radiation dose and contrast material without compromising image quality when compared with a standard 120 kVp CTA protocol.
